# Unmet Needs in Anticoagulant Therapy: Potential Role of Rivaroxaban

**DOI:** 10.14740/cr413w

**Published:** 2015-06-11

**Authors:** John W. Eikelboom, Stuart J. Connolly

**Affiliations:** a Population Health Research Institute, McMaster University and Hamilton Health Sciences, 237 Barton St. E., Hamilton, ON L8L 2X2, Canada

**Keywords:** Anticoagulation, Dabigatran, Rivaroxaban, Apixaban, Unmet needs

## Abstract

The new generation of non-vitamin K antagonist oral anticoagulants (NOACs) have been welcomed as a convenient alternative to warfarin. Three new oral anticoagulants, dabigatran etexilate, rivaroxaban and apixaban have been approved for the prevention of stroke and systemic embolism (SSE) in patients with atrial fibrillation (AF) and the prevention of venous thromboembolic events (VTEs) in patients who have undergone elective hip or knee replacement surgery. Dabigatran etexilate and rivaroxaban are also indicated for the treatment of VTE and the long-term prevention of recurrent deep vein thrombosis (DVT) and pulmonary embolism (PE). A fourth agent, edoxaban, has been successfully tested for several indications but is not yet approved for use in North America or Europe. Building on these successes, new trials are planned to address remaining unmet needs and knowledge gaps. This paper examines the unresolved issues in anticoagulant therapy with a focus on planned and ongoing trials.

## Introduction

For more than 60 years, vitamin K antagonists (VKAs), such as warfarin, have been the only oral anticoagulants available for clinical use. Warfarin is effective for the prevention and treatment of thrombosis but has drawbacks that limit its uptake and use [1]. Three recently introduced non-vitamin K antagonist oral anticoagulants (NOACs), dabigatran etexilate, rivaroxaban and apixaban, now offer clinicians and patients much-needed alternatives to warfarin. A fourth NOAC, edoxaban, has successfully completed testing for several indications but is not yet approved and is not further discussed in this paper.

NOACs have been successfully tested as alternatives to standard therapies in randomized trials involving more than 135,000 patients and are approved around the world for prevention of stroke and systemic embolism (SSE) in patients with atrial fibrillation (AF), prevention of venous thromboembolism (VTE) in patients who have undergone elective hip or knee replacement surgery; the initial and long-term treatment of VTE; and the prevention of atherothrombotic events after an acute coronary syndrome (ACS).

**Figure 1:**
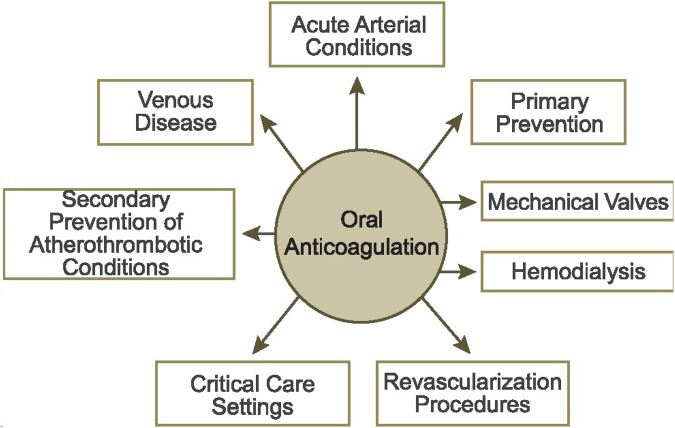
Clinical settings for oral anticoagulation. Clinical questions about the management of the NOACs exist in each of these settings.

Despite the established efficacy of NOACs for multiple indications, knowledge gaps remain (Fig. 1). To help bridge these gaps, new studies are planned or ongoing. This paper explores unmet needs in anticoagulant therapy and summarizes the ongoing rivaroxaban clinical trial program for both established (Table 1) and new indications (Table 2).

**Table 1:** Ongoing Studies With Rivaroxaban Addressing Unresolved Issues in Established Indications

Trial	Clinical question(s) to be addressed	Arms	Targeted enrollment
*Stroke prevention in patients with AF*			
VENTURE-AF NCT01729871	Does rivaroxaban need to be stopped prior to catheter ablation in patients with non-valvular AF?	Rivaroxaban 20 mg QD VKA (INR 2.0-3.0)	250
X-TRA NCT01674647	Does treatment with rivaroxaban regress LA thrombi in patients with non-valvular AF or atrial flutter?	Rivaroxaban 20 mg QD	60
*VTE treatment*			
EINSTEIN CHOICE NCT02064439	Can a lower dose (10 mg) of rivaroxaban be used for the long-term prevention of VTE, and is this or 20 mg rivaroxaban superior to ASA?	Rivaroxaban 10 mg QD Rivaroxaban 20 mg QD ASA 100 mg QD	2,850
EINSTEIN Junior NCT01684423	Can rivaroxaban be safely used in children?	Rivaroxaban (age and weight adjusted) Standard of care	50
Superficial vein thrombosis NCT01499953	Can rivaroxaban be used to treat SVT?	Rivaroxaban 20 mg QD	506
*Prevention of VTE*			
MARINER NCT02111564	Can rivaroxaban reduce the risk of post-hospital discharge symptomatic VTE in patients hospitalized for acute medical illness?	Rivaroxaban 10 mg QD or 7.5 mg QD (adjusted for creatine clearance) Placebo	8,000
EPCAT II NCT01720108	Is rivaroxaban more effective than ASA for extended prophylaxis of VTE?	Rivaroxaban 10 mg QD Fondaparinux 2.5 mg QD	3,436
*Therapeutic approaches for patients with coronary disease*			
GEMINI ACS 1 NCT02293395	Can rivaroxaban, in combination with a single antiplatelet agent, be used for secondary prevention in patients with ACS?	Study design not released at the time of publication	3,000
X-PLORER NCT01442792	Can rivaroxaban prevent thrombosis and related adverse ischemic events during elective PCI?	Rivaroxaban 20 mg QD Rivaroxaban 10 mg QD Rivaroxaban 10 mg QD + UFH 50 IU/kg bolus UFH 70-100 IU/kg bolus	106
PIONEER AF-PCI NCT01830543	Should rivaroxaban be used over warfarin in patients who have undergone PCI and are on antiplatelet therapy? If so, what is the optimal rivaroxaban dose and antiplatelet regimen for these patients?	Rivaroxaban 15 mg QD + P2Y12 inhibitor* Rivaroxaban 2.5 mg BID + DAPT VKA + DAPT	2,100

*P2Y12 inhibitor such as clopidogrel (75 mg QD). ACS: acute coronary syndrome; AF: atrial fibrillation; ASA: acetylsalicylic acid; DAPT: dual antiplatelet therapy; LA: left atrial; PCI: percutaneous coronary intervention; SVT: superficial vein thrombosis; UFH: unfractionated heparin; VKA: vitamin K antagonist; VTE: venous thromboembolic events.

**Table 2:** Ongoing Studies With Rivaroxaban in Potential New Indications

Trial	Clinical question(s) to be addressed	Arms	Estimated enrollment
*Secondary prevention of cardiovascular disease*			
COMPASS NCT01776424	Does rivaroxaban provide additional cardioprotective benefits to high-risk patients? Should rivaroxaban be used alone or in combination with ASA in patients with CAD or PAD?	Rivaroxaban 2.5 mg BID + ASA Rivaroxaban 5.0 mg BID ASA 100 mg QD	19,500
*Heart failure*			
COMMANDER-HF NCT01877915	Does adding low-dose rivaroxaban to optimal medical therapy improve outcomes in heart failure?	Rivaroxaban 2.5 mg BID + single or dual antiplatelet therapy Single or dual antiplatelet therapy	5,000
*Confirmed or suspected HIT*			
Heparin-induced thrombocytopenia NCT01598168	Can rivaroxaban be used to treat HIT?	Rivaroxaban 15 mg BID until HIT excluded, followed by rivaroxaban 20 mg QD	200
*Stroke prevention in patients with ESUS*			
NAVIGATE ESUS NCT02313909	Is rivaroxaban more effective than ASA atreducing the risk of recurrent stroke and systemic embolism in patients with a recent ESUS?	Rivaroxaban 15 mg QD ASA 100 mg QD	7,000
*Treatment of high-risk patients with non-disabling cerebrovascular events*			
TRACE NCT01923818	Should rivaroxaban be used over ASA in patients following TIA or minor ischemic stroke?	ASA 100 mg QD Rivaroxaban 5 mg Rivaroxaban 10 mg	3,700

ASA: acetylsalicylic acid; CAD: coronary artery disease; ESUS: embolic stroke of undetermined source; HIT: heparin-induced thrombocytopenia; PAD: peripheral artery disease; TIA: transient ischemic attack.

## Unresolved Issues in Established Indications

### Stroke prevention in patients with AF

Dabigatran, rivaroxaban and apixaban have been shown in the RE-LY, ROCKET-AF and ARISTOTLE trials, respectively, to be non-inferior or superior to warfarin, with similar or reduced rates of major bleeding [2-4]. All NOACs reduced intracranial bleeding, the most feared complication of warfarin therapy, and demonstrated favorable effects on mortality. Two related areas that require further study include AF patients undergoing procedures (restore sinus rhythm, device implantation or ablation), and those with thrombosis in the left atrium (LA) or left atrial appendage (LAA).

#### Cardioversion

Clinicians have extensive experience using warfarin in patients undergoing cardioversion. Data supporting the use of NOACs for patients undergoing cardioversion are limited to secondary analyses from the major registration trials and observational studies [5-7]. *Post-hoc* analyses of RE-LY (n = 1,270), ROCKET-AF (n = 143) and ARISTOTLE (n = 540) suggest NOACs are safe and effective for patients undergoing cardioversion, with similar rates of stroke and bleeding compared to warfarin [5-7]. However, the number of patients in these analyses was modest, particularly for rivaroxaban and apixaban.

Recently, the X-VeRT trial found rivaroxaban to be an effective and safe alternative to VKAs for the prevention of major cardiovascular (CV) events in patients with AF undergoing elective cardioversion (early or delayed strategy) [8]. In this trial, 1,500 patients scheduled for cardioversion were randomized to rivaroxaban 20 mg QD (15 mg in those with creatinine clearance 30 - 49 mL/min) or warfarin (INR 2 - 3). The primary efficacy outcome of composite of stroke, transient ischemic attack (TIA), non-central nervous system (non-CNS) systemic embolism (SE), myocardial infarction (MI) and CV death occurred in 0.51% and 1.02% of patients in the rivaroxaban and VKA arms, respectively. The primary safety outcome of major bleeding occurred in 0.6% and 0.8% of patients, respectively. The results of this trial suggest that rivaroxaban may allow for prompter cardioversion.

A similar study is planned with apixaban (NCT02100228), and another study is testing the utility of routine transesophageal echocardiogram (TEE) in patients treated with dabigatran undergoing cardioversion (NCT01593150).

#### Catheter ablation

For patients undergoing catheter ablation, anticoagulation with warfarin has been the standard of care. Anticoagulation therapy is generally interrupted 5 days prior to the procedure and recommenced post-procedure with administration of bridging low molecular weight heparin (LMWH) during the period of interruption. A meta-analysis comparing dabigatran to warfarin in patients undergoing catheter ablation found a similar incidence of thromboembolic events and major bleeding compared to warfarin, with low event rates overall [9]. Similar experience has been reported with rivaroxaban [5]; however, no data are available for apixaban in this setting. Additional data are needed to strengthen the rationale to use NOACs in patients undergoing ablation and to determine the optimal peri-procedural management strategy (continued vs. interrupted use of NOAC, timing of stopping and restarting, need for TEE prior to the procedure, etc.).

VENTURE-AF (NCT01729871) is an open label, multicenter trial comparing uninterrupted rivaroxaban with usual care in patients with persistent or paroxysmal non-valvular AF scheduled to undergo their first catheter ablation [10]. Two hundred and fifty patients are being randomized to receive uninterrupted rivaroxaban 20 mg QD or warfarin for at least 28 days prior to catheter ablation, followed by 30 ± 5 days of treatment post-procedure. All patients will receive intravenous heparin during catheter ablation. Prior to catheter ablation, patients are required to demonstrate sufficient anticoagulation during the 3 weeks before randomization or undergo a TEE. This trial is not powered for efficacy outcomes and the primary outcome is the incidence of major bleeding events 30 ± 5 days following the ablation procedure. This study has been completed and is awaiting results.

Studies investigating the safety and efficacy of uninterrupted dabigatran (RE-CIRCUIT; NCT02348723) or apixaban (AXAFA; NCT02227550) in patients with AF undergoing catheter ablation are also planned. The results of these trials are expected in 2016 and 2017, respectively.

#### Device implantation

Current guidelines recommend warfarin interruption and bridging therapy with heparin around the time of device implantation [11]. This approach has been superseded by the results of the recently published BRUISECONTROL trial which demonstrated superior safety of continuous compared with interrupted warfarin therapy at the time of pacemaker or ICD surgery [12].

There are limited data for NOACs in patients undergoing device implantation and it is unclear if an NOAC must be stopped prior to the procedure and whether bridging with heparin is required [13]. Observational data suggest uninterrupted dabigatran during device implantation is safe, with no serious bleeding or thromboembolic events reported [14]. This approach is being further investigated in the ongoing BRUISECONTROL2 (NCT01675076) randomized controlled trial (RCT).

#### LA/LAA thrombosis

The identification of an LA or LAA thrombus poses a challenging management problem in patients with AF. Conventional treatment involves the use of heparin or LMWH for at least 5 days overlapped by a VKA. Trials of NOACs for initial treatment of VTE used higher doses of rivaroxaban (20 mg QD) and apixaban (10 mg BID) for the first 1 - 3 weeks in order to ensure adequate suppression of coagulation [15, 16]. It is unclear whether higher doses are also required in the initial management of cardiac thrombus. X-TRA (NCT01839357) is an observational study evaluating the use of rivaroxaban 20 mg QD in 60 patients with LA/LAA thrombus. The primary outcome is resolution of LA/LAA thrombus after 6 weeks of treatment, as assessed by TEE. This study has been completed and is awaiting results.

#### VTE treatment

Dabigatran, rivaroxaban and apixaban have demonstrated non-inferiority to conventional therapy (subcutaneous enoxaparin followed by a VKA) for the initial and long-term treatment of VTE, with similar or superior safety profiles [15-18]. Ongoing clinical trials are exploring the optimal dose of rivaroxaban for extended VTE prevention and are investigating whether rivaroxaban can be used in patients with superficial vein thrombosis (SVT) and children with VTE.

#### Extended duration treatment of VTE

Extended treatment with dabigatran, rivaroxaban and apixaban is effective at reducing the risk of recurrent VTE [16, 17, 19]; however, the optimal dose and duration of extended treatment is not clear. In AMPLIFY-EXT, patients who previously received 6 - 12 months of anticoagulant therapy were randomized to extended treatment with apixaban at the standard dose (5 mg BID) or lower dose (2.5 mg BID) [20]. The lower dose of apixaban was found to be as effective as the standard dose, with rates of bleeding similar to placebo. Standard doses of rivaroxaban and dabigatran were investigated in their respective extension trials. It remains unresolved whether a lower dose would preserve efficacy while reducing the risk of bleeding during extended treatment.

EINSTEIN-CHOICE (NCT02064439) is testing the efficacy of a reduced dose of rivaroxaban for extended duration VTE prevention. This trial will randomize 2,850 patients who have received 6 - 12 months anticoagulant therapy to rivaroxaban 10 mg QD, rivaroxaban 20 mg QD or acetylsalicylic acid (ASA) 100 mg QD. In addition to informing on the safety and efficacy of a lower dose rivaroxaban strategy, this trial will also provide information on whether rivaroxaban is superior to ASA for long-term VTE prevention.

#### Pediatric thrombosis

Over the past 15 years, the frequency of VTE diagnosis in hospitalized children has increased 3- to 10-fold, from 0.3 - 28.8 cases per 10,000 admissions between 1992 and 2005 to 34 - 58 cases per 10,000 admissions between 2001 and 2007 [19, 21, 22]. Despite the increased burden of pediatric thrombosis, high quality evidence from RCTs concerning the optimal dose, efficacy and safety of commonly used anticoagulants, including unfractionated heparin (UFH), LMWH, warfarin and the NOACs is lacking. To help address this gap, EINSTEIN Junior (NCT01684423) is investigating the pharmacokinetic/pharmacodynamic (PK/PD) properties, safety and efficacy of rivaroxaban in children with various manifestations of venous thrombosis. In this phase II study, children aged 6 - 17 are randomized to age- and body weight-adjusted rivaroxaban or standard of care for 30 days. The primary outcome is major and clinically relevant non-major bleeding. Study completion is expected in September 2015. Pediatric trials are also underway for dabigatran (NCT01895777) and apixaban (NCT01707394).

#### Superficial vein thrombosis

Superficial vein thrombosis affects approximately 3-11% of the general population (annual incidence is not known) and is associated with an increased risk of VTE [23, 24]. Anticoagulants reduce acute symptoms, prevent extension, and reduce the risk of progression to VTE [25]. Fondaparinux administered parenterally for 45 days is effective for the treatment of SVT, but must be injected, making it an inconvenient therapy for patients. The superficial vein thrombosis treated with rivaroxaban versus fondaparinux trial (NCT01499953) is randomizing 506 patients to rivaroxaban 10 mg QD or fondaparinux 2.5 mg QD for 45 days. The primary efficacy outcome is death from any cause and the primary safety outcome is major bleeding events. Study completion is expected in March 2017.

#### Prevention of VTE

Prophylactic anticoagulation to prevent VTE is standard of care following total hip replacement (THR) or total knee replacement (TKR) surgery. Dabigatran, apixaban and rivaroxaban are approved for these indications based on comprehensive clinical trial programs that collectively randomized more than 34,000 patients [26-38].

Emerging evidence indicates that ASA is also effective for VTE prophylaxis in patients undergoing major orthopedic surgery [39, 40]. The American College of Chest Physicians (ACCP) and American Academy of Orthopaedic Surgeons (AAOS) guidelines recommend ASA as an effective alternative to anticoagulants for VTE prevention, but its efficacy and safety relative to the NOACs is unknown [41-43]. The EPCAT II trial (NCT01720108) is randomizing 3,436 patients undergoing TKR or THR to either rivaroxaban 10 mg QD or ASA 81 mg QD for 9 days (TKR) or 30 days (THR). All patients will receive initial treatment with rivaroxaban for 5 days after surgery, prior to randomization. The primary efficacy outcome is symptomatic VTE and the primary safety outcome is major or clinically relevant non-major bleeding. The trial is expected to finish in December 2017.

#### Prevention of VTE in the medically ill

Patients who have been hospitalized for the treatment of acute medical illnesses are at high risk for the development of VTE after hospital discharge [44]. Extended duration rivaroxaban has been found to reduce the risk of VTE in patients hospitalized for an acute medical illness [45]. To determine if rivaroxaban can prevent symptomatic VTE in post-hospital discharge patients, the MARINER study (NCT02111564) will randomize 8,000 high-risk, medically ill patients to rivaroxaban 10 mg QD (7.5 mg QD if creatinine clearance is 30 - 49 mL/min) or placebo. The primary outcomes are time from randomization to the first occurrence of symptomatic VTE, VTE-related death or major bleeding. Study completion is expected in February 2017.

### Therapeutic approaches for patients with coronary disease

Following an ACS, patients are at an increased risk for recurrent CV events and require long-term dual antiplatelet therapy (DAPT; ASA plus P2Y12 inhibitor). Interest in the use of NOACs in this setting has increased as these patients also have persistently elevated markers of coagulation activation which is associated with an increased risk of recurrent major adverse CV events [46, 47].

The safety and efficacy of dabigatran and apixaban for the secondary prevention of ACS was investigated in the phase II RE-DEEM and phase III APPRAISE-2 trials, respectively [48, 49]. These trials found an increased risk of bleeding, with no benefit on the reduction in risk of recurrent ischemic events. Of note, APPRAISE-2 was halted early and no phase III trial with dabigatran was initiated in this setting.

In contrast, rivaroxaban (2.5 mg BID and 5 mg BID in addition to ASA alone or ASA plus a thienopyridine) was found to significantly reduce the composite of death from CV causes, MI, or stroke [50]. Based on these findings, rivaroxaban 2.5 mg BID was approved in Europe for the prevention of atherothrombotic events in patients after ACS. In follow-up to this study, the benefits of rivaroxaban in combination with single antiplatelet treatment for long-term secondary prevention after ACS will be investigated in the phase II GEMINI ACS 1 trial (NCT02293395). This trial will involve 3,000 patients, and if successful, the phase III trial, GEMINI ACS 2, will be undertaken to validate the results of the phase II study.

Patients with ACS routinely undergo percutaneous coronary intervention (PCI), with standard anticoagulant therapy consisting of UFH, enoxaparin or bivalirudin [51-53]. No studies have investigated the use of NOACs in patients undergoing PCI with stenting. As such, the question remains unanswered whether the NOACs can be used in these patients who also have an indication for DAPT.

#### Elective PCI

The X-PLORER trial (NCT01442792) is investigating whether rivaroxaban can be used without interruption during elective PCI in patients treated with DAPT. This trial is randomizing 106 patients to one of four arms: UFH (70 - 100 IH/kg bolus); rivaroxaban (10 mg single dose); rivaroxaban (20 mg single dose); or rivaroxaban (10 mg single dose) plus UFH (50 IU/kg bolus). Although severely underpowered, this trial will provide some information on the efficacy of rivaroxaban compared with heparin for prevention of catheter thrombosis. Earlier trials have shown the selective factor Xa inhibitor, fondaparinux, to be associated with an increase in catheter thrombosis compared with enoxaparin [27, 28]. The increased risk of catheter thrombosis with fondaparinux was prevented by the use of a small dose of heparin at the time of the procedure [53, 54]. It is unclear whether rivaroxaban might also be associated with increased catheter thrombosis and whether the use of a small heparin bolus may obviate this risk.

#### PCI in patients with an indication for anticoagulant therapy

The optimal antithrombotic therapy post-PCI with stenting in patients who require chronic oral anticoagulation is uncertain [55]. DAPT is required to prevent stent thrombosis but the combination of a VKA and DAPT greatly increases the risk of bleeding [56, 57]. The WOEST trial demonstrated that the combination of warfarin and clopidogrel reduces the risk of bleeding without increasing thrombotic events as compared to triple therapy with warfarin + clopidogrel + ASA in patients undergoing coronary artery stenting [58].

NOACs are an attractive alternative to warfarin in patients with an indication for anticoagulation who undergo PCI with stenting because they produce less bleeding. Additionally, both rivaroxaban and apixaban (factor Xa inhibitors) reduce the risk of stent thrombosis in patients with ACS. However, the efficacy of NOACs in patients with an indication for both DAPT and therapeutic dose anticoagulation has yet to be formally tested [49, 50].

The RE-DUAL PCI trial (NCT02164864) is testing the efficacy and safety of dabigatran post-PCI. This trial will randomize 8,520 patients with AF who undergo PCI with stenting to dabigatran dual therapy (dabigatran + clopidogrel or ticagrelor) or warfarin triple therapy (warfarin + ASA + clopidogrel or ticagrelor). Additionally, PIONEER AF-PCI (NCT01830543) is testing the safety of rivaroxaban in patients with non-valvular AF who have undergone PCI. This trial is randomizing 2,100 patients to: 12 months of rivaroxaban 15 mg QD + P2Y12 inhibitor; 1, 6 or 12 months of rivaroxaban 2.5 mg BID + DAPT, followed by rivaroxaban 15 mg + ASA for a total of 12 months; or 1, 6 or 12 months of VKA + DAPT, followed by VKA + ASA for a total of 12 months. Final data collection for this study is expected in August 2016. A similar trial is also planned with apixaban.

## New Indications in Areas of Unmet Need

### Secondary prevention of cardiovascular disease

Coronary artery disease (CAD) is the most common cause of CV disease, responsible for 7.3 million deaths annually [59, 60]. Patients with established CV disease are at increased risk of MI, stroke, and CV death [61]. ASA is effective for CV prevention, but despite its routine use, patients continue to have platelet and coagulation activation and high rates of CV events [62-64]. Previous trials aimed at improving therapy by replacing ASA with clopidogrel, combining ASA with a second antiplatelet agent or combining ASA with warfarin have produced disappointing results [65-67]. Rivaroxaban is effective for prevention of MI, stroke or CV death in patients with ACS and rivaroxaban-based therapy has the potential to improve efficacy for CV prevention compared with ASA alone.

**Figure 2:**
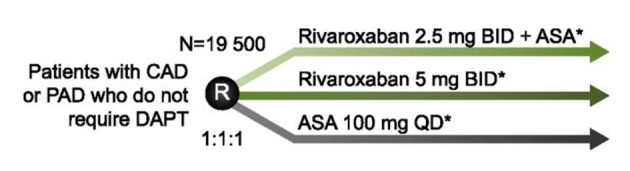
Study design of COMPASS. To assess the safety and efficacy of rivaroxaban for the prevention of MI, stroke, and CV death, patients with coronary artery disease (CAD) or peripheral artery disease (PAD), who do not require dual antiplatelet therapy (DAPT), are randomized to rivaroxaban 2.5 mg BID + acetylsalicylic acid (ASA) 100 mg QD; rivaroxaban 5 mg BID; or ASA 100 mg QD. *Patients in each arm are also randomized to pantoprazole 40 mg QD or placebo to investigate if safety can be improved by the addition of a proton pump inhibitor.

The COMPASS trial (NCT01776424) is randomizing 21,400 patients with CAD or peripheral artery disease (PAD) to receive rivaroxaban 2.5 mg BID + ASA 100 mg QD, rivaroxaban 5 mg BID or ASA 100 mg QD for prevention of MI, stroke or CV death (Fig. 2). A second factorial randomization is testing whether pantoprazole 40 mg QD compared with placebo can prevent a composite of gastrointestinal complications and thereby improve the safety of antithrombotic therapy in this population. COMPASS is also testing whether rivaroxaban-based therapy is superior to aspirin for prevention of graft failure following coronary artery bypass grafting (CABG) surgery. A substudy, COMPASS MIND, is examining whether rivaroxaban can prevent covert brain ischemia and related cognitive decline. Study completion is expected in February 2018.

### Heart failure

**Figure 3:**
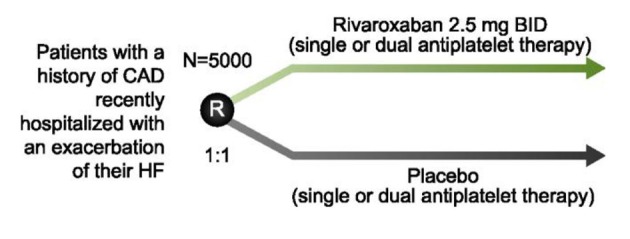
Study design of COMMANDER-HF. To determine whether rivaroxaban reduces the risk of MI, stroke or death, patients with a history of coronary artery disease (CAD) or those recently hospitalized for an exacerbation of their heart failure (HF) are randomized to rivaroxaban 2.5 mg BID or placebo in addition to their current standard of care.

Heart failure (HF) is one of the leading causes of hospitalization and is associated with an annual mortality rate of up to 50% in Canada [68, 69]. Patients with HF are at increased risk of thromboembolic complications such as stroke, myocardial ischemia and death [70-72]. Trials with warfarin have produced modest reductions in CV events at the cost of substantial increases in bleeding [72-76]. No large scale clinical trials have investigated the NOACs in this setting. The COMMANDER-HF trial (NCT01877915) is randomizing 5,000 patients with a history of CAD, recently hospitalized for an HF exacerbation, to either rivaroxaban 2.5 mg BID or placebo on a background of optimal medical therapy, including single or dual antiplatelet therapy (Fig. 3). The primary outcome is the composite of MI, stroke, or all-cause mortality. This study is expected to be completed in April 2017.

### Confirmed or suspected HIT

Type II heparin-induced thrombocytopenia (HIT) is triggered by an immune response to heparin and can lead to limb- or life-threatening thromboembolic complications. Although HIT is uncommon, suspected HIT warrants discontinuation of heparin and treatment with an alternative anticoagulant [77]. Non-heparin anticoagulants: lepirudin, argatroban and danaparoid, are recommended for the treatment of HIT or suspected HIT but are expensive and require parenteral administration. An oral alternative would provide an attractive option that would be more convenient and less expensive.

The rivaroxaban for treatment of patients with suspected or confirmed HIT trial (NCT01598168) is examining the incidence of new symptomatic venous and arterial thromboembolism in 200 patients with intermediate or high clinical probability of HIT (4T’s score ≥ 4). These patients are being treated with rivaroxaban 15 mg BID until HIT is excluded or the platelet count has recovered. After recovery of the platelet count, patients with confirmed HIT receive rivaroxaban 20 mg QD until day 30.

### Stroke prevention in patients with embolic stroke of undetermined source (ESUS)

Strokes resulting from unknown sources comprise approximately 25% of all ischemic strokes, yet there remains limited evidence for secondary prevention strategies [78]. Randomized trials investigating oral anticoagulants for secondary prevention in patients with ESUS are warranted. NAVIGATE ESUS (NCT02313909) will evaluate whether rivaroxaban 15 mg QD is superior to aspirin 100 mg QD in reducing the risk of recurrent stroke and systemic embolic events in 7,000 patients with a recent ESUS. The results of this trial are expected in 2018. A similar trial is also planned with dabigatran (RE-SPECT ESUS; NCT02239120); this study is currently recruiting patients with final data collection planned for November 2017.

### Treatment of high-risk patients with non-disabling CV events

Patients who experience a TIA or non-disabling ischemic stroke are at an increased risk of early, recurrent stroke [79]. Early administration of ASA is recommended to improve outcomes in these patients but is only modestly effective [79-83]. Emerging evidence suggests that early intensive therapy with DAPT is more effective than ASA alone and does not increase the risk of bleeding [84]. Guidelines recommend against the use of heparin and warfarin because of concerns about an increased risk of intracranial bleeding [82, 85]. The NOACs are an attractive option in patients with stroke because they are at least as effective as warfarin for prevention of thromboembolic events and are associated with a substantially lower risk of intracranial bleeding.

Rivaroxaban is being investigated for the treatment for non-disabling CV events in the Chinese TRACE study (NCT01923818). This trial is randomizing 3,700 patients to 30 days of ASA 100 mg, rivaroxaban 5 mg, or rivaroxaban 10 mg. The primary outcome is recurrent stroke at 90 days.

## Summary and Conclusion

Recent years have seen rapid advancement in new anticoagulant therapies for patients at risk of thromboembolic events. The NOACs have shown to be at least as effective as standard therapies across a broad range of indications, but many unanswered questions and unmet needs remain. New trials are dedicated to addressing these unmet needs in populations spanning established and potential new indications.

Rivaroxaban is currently being tested in clinical trials involving more than 54,000 patients and studies with dabigatran and apixaban are also ongoing. As data from these trials become available, we will begin to bridge the existing knowledge gaps and gather insight on optimal patient care in diverse clinical settings.
